# MIMO Radar Parallel Simulation System Based on CPU/GPU Architecture

**DOI:** 10.3390/s22010396

**Published:** 2022-01-05

**Authors:** Gaogao Liu, Wenbo Yang, Peng Li, Guodong Qin, Jingjing Cai, Youming Wang, Shuai Wang, Ning Yue, Dongjie Huang

**Affiliations:** School of Electronic Engineering, Xidian University, Xi’an 710071, China; wbyang_46@stu.xidian.edu.cn (W.Y.); penglixd@xidian.edu.cn (P.L.); gdqin@mail.xidian.edu.cn (G.Q.); jjcai@mail.xidian.edu.cn (J.C.); ymwang_8@stu.xidian.edu.cn (Y.W.); shwang@stu.xidian.edu.cn (S.W.); nyue@stu.xidian.edu.cn (N.Y.); huangdj@stu.xidian.edu.cn (D.H.)

**Keywords:** graphics processing unit (GPU), central processing unit (CPU), parallel processing, multiple-input multiple-output radar

## Abstract

The data volume and computation task of MIMO radar is huge; a very high-speed computation is necessary for its real-time processing. In this paper, we mainly study the time division MIMO radar signal processing flow, propose an improved MIMO radar signal processing algorithm, raising the MIMO radar algorithm processing speed combined with the previous algorithms, and, on this basis, a parallel simulation system for the MIMO radar based on the CPU/GPU architecture is proposed. The outer layer of the framework is coarse-grained with OpenMP for acceleration on the CPU, and the inner layer of fine-grained data processing is accelerated on the GPU. Its performance is significantly faster than the serial computing equipment, and satisfactory acceleration effects have been achieved in the CPU/GPU architecture simulation. The experimental results show that the MIMO radar parallel simulation system with CPU/GPU architecture greatly improves the computing power of the CPU-based method. Compared with the serial sequential CPU method, GPU simulation achieves a speedup of 130 times. In addition, the MIMO radar signal processing parallel simulation system based on the CPU/GPU architecture has a performance improvement of 13%, compared to the GPU-only method.

## 1. Introduction

Multiple-input multiple-output (MIMO) radar is defined broadly as a radar system employing multiple transmit waveforms and having the ability to jointly process signals received at multiple receive antennas [1]. Elements of MIMO radar transmit independent waveforms result in an omnidirectional beampattern or create diverse beampatterns by controlling correlations among transmitted waveforms [2]. In [3], it is observed that MIMO radar has more degrees of freedom than systems with a single transmit antenna. These additional degrees of freedom support flexible time-energy management modes [4], lead to improved angular resolution [5,6]. MIMO radar handles slow moving targets by exploiting Doppler estimates from multiple directions which allows MIMO radar to have a low probability intercept (LPI) [7] and support high-resolution target localization [8]. In addition, MIMO radar has the characteristics of significantly improved radar speed resolution and radar search ability, radar anti-jamming and anti-clutter performance, and the angular resolution [9]. Due to its abovementioned advantages, MIMO radar is widely used in remote sensing, navigation, weather forecast, resource detection, and other fields [10,11].

From the perspective of MIMO radar working mode, MIMO radar is mainly divided into three categories: one is time division multiplexing (TDM), the other is frequency division multiplexing (FDM), and the rest is code division multiplexing (CDM). Time division multiplexing MIMO radar transmits signals from one element per time slot. The algorithm of time division MIMO radar includes the windowing algorithm, moving target indication (MTI), moving target detection (MTD) algorithm, transmit beam and receive beam forming algorithm, and constant false alarm rate algorithm. The frequency division multiplexing MIMO radar is used to detect the target by setting the signals of different transmit array elements. The transmitting signals among the array elements are orthogonal to each other, and the receiving signals are matched and filtered to separate different transmitting signals. Frequency-division MIMO radar algorithms include digital beam forming (DBF) algorithm, pulse synthesis algorithm, MTI, and MTD algorithm [12]. CDM offers more degrees of freedom than TDM, which results in a more flexible design of the transmission sequences. On the other hand, a time division multiplexing MIMO radar requires less complex hardware. This is especially important for radars applied to automobiles, as they have to be produced at low cost [13,14]. The transmission waveform of the time division MIMO radar can use the chirp signal, and the signals transmitted by different transmission array elements can be distinguished according to time. The algorithm processing is simple, so time division MIMO radar was adopted for processing in this paper.

With the development of MIMO radar technology, MIMO radar is used in high-resolution, multitarget tracking, virtual array elements, and multimode. For example, the study of MIMO synthetic aperture radar (SAR) is a fascinating research field. The concept of MIMO SAR was first introduced in [15]. With the increased resolution of SAR systems and the demand for 3D applications [16], the objects of SAR raw data simulation change from point targets or surface targets into natural 3D terrain, which causes the rapid growth of computational time. Therefore, it is necessary to improve computational efficiency for the wide application of MIMO SAR.

A combination of programmable logic gate arrays and digital signal processing based on hardware boards is adopted [17]. These two methods are expensive and have poor scalability. Another method is to use the central processor for parallel computing [18]. The central processing unit (CPU) can handle huge amounts of data, but there are many shortcomings, and this method is difficult to develop [19]. Among the known published studies, the effect of the parallelization for the field programmable gate array (FPGA) module can be better than CPU [20,21]. However, the former has high design complexity, and the absolute calculation speed is not high. Although the methods described in these papers are optimal for general data volume, they may not be the method of choice otherwise. Therefore, it is desired to provide a more efficient method to simulate the MIMO radar signal processing.

As GPU computing power continues to enhance, using GPU to accelerate radar signal processing algorithms has the advantages of improving computing speed and reducing development costs. Therefore, in view of the extremely strong computing power of GPU, it will be more commonly used in various types of radar signal processing algorithms in the future development of science and technology. In order to improve the efficiency of MIMO radar signal processing simulation, a CPU-oriented method and a GPU-oriented method are used in this paper. The CPU-oriented approach mainly refers to parallel simulation on the CPU platform, such as multicore open multiprocessing (OpenMP) [22], multi-CPU message passing interface (MPI), and multimachine grid computing. The acceleration effect of these methods is proportional to the number of CPUs. The CPU hardware device cluster uses multiple CPUs for parallel calculation, but the cost of deploying the CPU hardware device cluster is high, resulting in high parallel simulation costs. Acceleration strategies may rely more on computer platforms than fast algorithms. As is known to all, the CPU clock frequency has not increased significantly today. Multicore CPU has become a new development direction.

A GPU-oriented method has realized parallel simulation of large-scale cores on the GPU platform, and, especially, general-purpose computing based on GPU has also become a very attractive development direction in recent years [23]. Early GPUs were mainly used for image processing, while modern GPUs are equipped with general-purpose programming interfaces such as NVIDIA’s CUDA (does not require programmers to master a lot of graphics knowledge), which is especially suitable for massively parallel numerical calculation [24]. How to make full use of GPU to improve the efficiency of MIMO radar signal processing has become a hot topic in recent years. Compared with the CPU method, the GPU method achieves a speedup of dozens to hundreds of times, and it is an efficient and low-cost solution for massive data MIMO radar signal processing.

However, in the GPU-based MIMO simulation, the CPU is often ignored as a computing resource. Normally, the CPU core remains idle, while the GPU core is busy with calculations. Heterogeneous CPU/GPU computing seems to be the best solution to further improve simulation efficiency [25], because an increasing trend is to use multiple computing resources (usually heterogeneous) as the only computing resources in the system. Heterogeneous simulation is a hybrid simulation that implements multicore CPU parallelism and large-core parallelism on the GPU. Because almost all existing ordinary computers are shared memory multicore systems, they can be easily upgraded to GPU/CPU platforms. Therefore, it is very meaningful to implement the heterogeneous parallel signal processing algorithm flow of time division MIMO radar on the GPU/CPU platform [26]. Reference [27] proposed a hybrid CPU–GPU multilevel preconditioner with a moderate memory footprint for solving a sparse system of equations resulting from finite element method (FEM) using higher-order elements. Reference [28] presented a parallel high-efficiency video-coding (HEVC) intraprediction algorithm for heterogeneous CPU+GPU systems. Reference [29] presented a full realization of the higher-order method of moments (HMoM) with a parallel out-of-core LU solver on GPU/CPU platform. An SAR raw data simulation method based on multicore SIMD processor and multi-GPU deep collaborative computing was proposed [26].

In this paper, a GPU-based time division MIMO radar signal processing algorithm flow is proposed. Compared with the previous MIMO radar signal processing algorithms, this method has the following characteristics. (1) We propose a time division MIMO radar signal processing algorithm based on GPU parallel acceleration. (2) In order to improve processing performance, we propose an improved GPU-based time division MIMO radar signal processing algorithm, which simplifies the process of the signal processing algorithm and speeds up the processing remarkably. (3) In order to enable the CPU to participate in the operation, we propose a parallel acceleration method of CPU based on OpenMP technology in combination with CUDA stream operation. The method uses pipeline operation to make the time division MIMO radar signal processing algorithm meet the real-time requirements, and further improves the calculation efficiency. The simulation results show that, compared with the traditional CPU-based time division MIMO radar signal processing algorithm flow, the proposed GPU-based time division MIMO radar signal processing algorithm has better performance, and each GPU processing algorithm is better than CPU processing. The processing efficiency of a single-core CPU has been increased by more than 50 times, and GPU computing with improved algorithms has achieved 130 times acceleration.

The rest of the article is structured as follows. Section 2 briefly introduces the basic principle of MIMO radar, the echo model, and the signal processing flow of time division MIMO radar. Section 3 mainly introduces time division MIMO radar algorithm optimization and GPU acceleration. The experimental results and optimization analysis are discussed in Section 4, and finally a conclusion is drawn.

## 2. MIMO Radar Signal Processing Algorithm

### 2.1. Basic Principles of MIMO Radar

**Figure 1:**
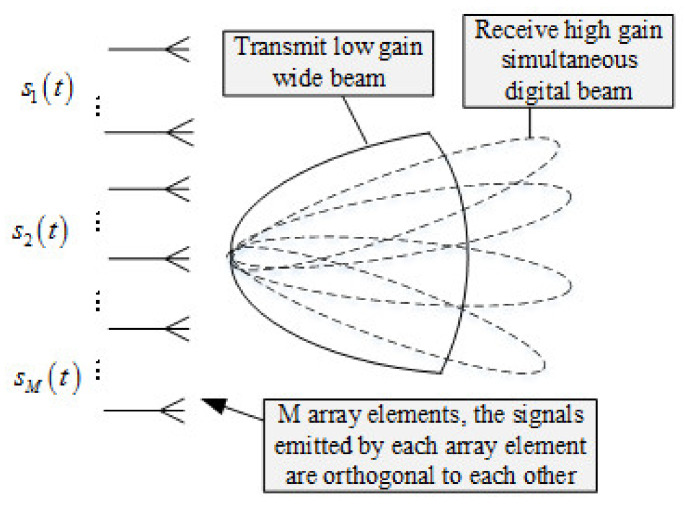
Basic principles diagram of MIMO radar.

The basic principles diagram of MIMO radar is shown in Figure 1. The transmitting terminal of the MIMO radar is composed of *M* transmit arrays. By controlling each digital transceiver unit at the transmitting terminal, the transmitting arrays transmit mutually orthogonal or partially orthogonal signal waveforms. The transmitted signal waveforms cannot be superimposed in the air to synthesize narrow beams with high gain while synthesizing wide beams with low gain in space [30]. However, the MIMO radar receives the target’s echo signal at the antenna receiving terminal, then uses digital beam-forming (DBF) technology to accumulate in the spatial domain, and finally form multiple high-gain narrow beams at the same time. Meanwhile, it can synthesize the receive and transmit beams with different directions by changing the digital beam coefficients.

### 2.2. MIMO Radar Echo Model

**Figure 2:**
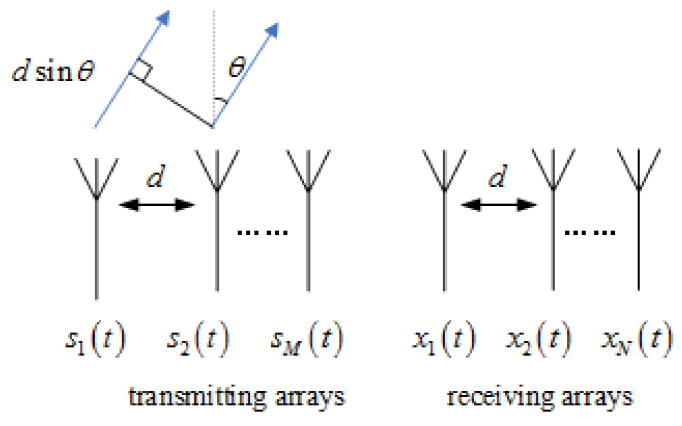
Schematic diagram of MIMO radar target transceiver.

Figure 2 shows the schematic diagram of MIMO radar transmission and reception. The number of transmitting and receiving arrays are *N* and *M*, respectively. They are arranged with equidistant lines. The array element interval is *d*, and the signals emitting by each array element are $s_{1}\left(t\right),s_{2}\left(t\right),\ldots ,s_{M}\left(t\right)$. They are orthogonal to each other [31].

Under far-field conditions, it is assumed that the angle between the target and the antenna element is *θ*. At the same time, taking the rightmost array element as a reference, due to transmission attenuation and time delay, the signal $s_{m}\left(t\right)$ sent by the *m*th arrays will become

(1) $$p_{m}\left(t\right)=\alpha_{1}s_{m}\left(t-\tau -\tau_{m}\right)=\alpha_{1}s_{m}\left(t-\tau \right)e^{-j\phi_{m}}$$

where $\alpha_{1}$ indicates the amplitude attenuation of the signal reaching the target. τ=R/c represents the time required for the signal emitted by the reference array element to propagate to the target. $\tau_{m}$ is the delay of the m array relative to the reference array to reach the target [32]. $\phi_{m}$ is the phase delay corresponding to $\tau_{m}$. c is the speed of light, and *R* is the distance between the reference array and the target. From the above isometric line array structure, it can be seen that between two adjacent array elements, the right array element is dsinθ more than the left array element from the target, so there is

(2) $$\tau_{m}=\frac{\left(m-1\right)d\sin\theta }{c}$$

(3) $$\phi_{m}=\frac{2\pi \left(m-1\right)d\sin\theta }{\lambda }$$

Assuming that the amplitude attenuation of each array’s transmitting signal to the target is $\alpha_{1}$ and all transmitting signals are narrow-band, the combined signal at the target can be written as

(4) $$\begin{array}{l} p(t)=\sum_{m=1}^{M_{t}}p_{m}\left(t\right)=\alpha_{1}\sum_{m=1}^{M_{t}}s_{m}\left(t-\tau \right)e^{-j\phi_{m}}\\ =\alpha_{1}\sum_{m=1}^{M_{t}}s_{m}\left(t\right)e^{-j\phi_{m}} \end{array}$$

The combined signal at the target will propagate to each receiving array after being reflected by the target. In a similar way, the amplitude attenuation of the reflected signal reaching each receiving array is $\alpha_{2}$. Similarly, the echo received by the *n* array element is

(5) $$\begin{array}{l} x_{n}\left(t\right)=\alpha_{2}p\left(t-\tau -\tau_{n}\right)+n_{n}\left(t\right)\\ =\alpha_{2}p\left(t\right)e^{-j\frac{2\pi \left(n-1\right)d\sin\theta }{\lambda }}+n_{n}\left(t\right)\\ =\alpha_{2}e^{-j\frac{2\pi \left(n-1\right)d\sin\theta }{\lambda }}\partial_{r}^{T}\left(\theta \right)S\left(t\right)+n_{n}\left(t\right) \end{array}$$

where $n_{n}\left(t\right)$ is the Gaussian white noise received by the *n* array, and $\vartheta_{r}$ represents receiving the steering vector [33,34].

### 2.3. Time Division MIMO Radar Processing Flow

**Figure 3:**
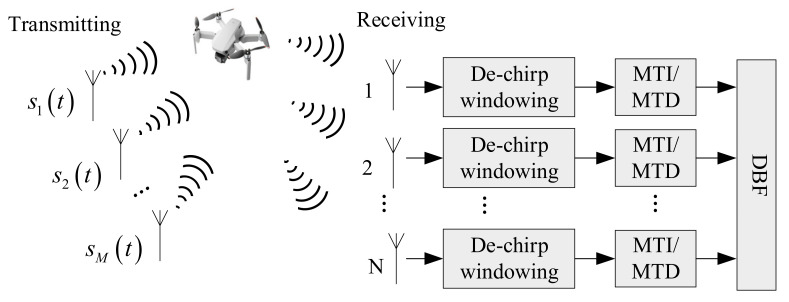
Flow chart of time division MIMO radar signal processing.

The time division multiplexing MIMO radar has *M* transmitting antenna array elements and *N* receiving antenna array elements, and its corresponding virtual antenna array is a uniform linear array, and it has *MN* antenna arrays, which are numbered in order from the array element to the *MN* antenna element according to the virtual position of the space. It transmits signals from one array per time slot. Through specific time division multiplexing timing of the transmitting antenna element and the receiving antenna element, the virtual antenna array receives signals according to the first element, the second element, the third element, the fourth element,…, the *MN*th element, which can distinguish the signals emitted by different transmitting arrays according to time. The time division MIMO radar signal processing flow chart is shown in Figure 3.

Under the time division MIMO system working mode, the basic process of echo signal processing is as follows:

1. Dechirp processing is performed on the received echo signal. Under the condition of ensuring the same resolution, the bandwidth of the signal can be greatly reduced, and the broadband signal is converted into a single frequency signal.
2. In order to suppress clutter and improve detection performance, we need advanced moving target indication (MTI) before detection, mainly to eliminate clutter and stationary targets. Moving target detection (MTD) processing is to perform fast Fourier transform (FFT) or FIR filtering on the data of the same distance units with different pulse repetition periods to eliminate the effects of clutter.
3. Simultaneous digital multibeam-forming (receiving beam-forming) of the *M* echo signals of the receiving channel to obtain high-gain receiving beams pointing in *k* directions. We reasonably set the weighting factors of the receiving steering vectors, which can flexibly control the receiving beam’s direction, so that the receiving beam is pointed to the space detection area of interest.

## 3. Time Division MIMO Radar Algorithm Optimization and GPU Acceleration

### 3.1. Improved Time Division MIMO Radar Processing Flow

It is also difficult to meet real-time requirements using GPU for processing, so the algorithm needs to be improved. In this process, windowing, MTI, MTD algorithm, and Doppler phase compensation are combined into one module for processing.

**Figure 4:**
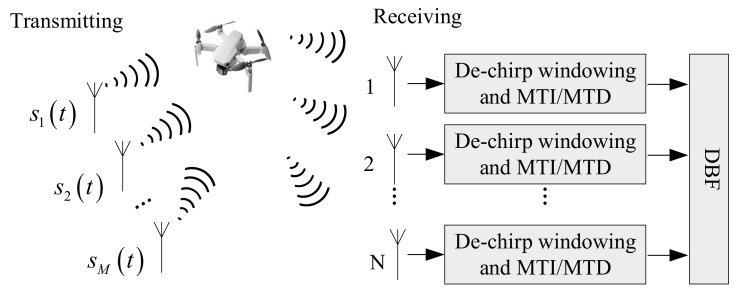
Improved time division MIMO radar signal processing flowchart.

Windowing minimizes spectrum leakage, resulting in low sidelobe. The MTI window improves the distance–dimensional main and sidelobe ratio characteristics, and the MTD window improves the Doppler-dimensional main and sidelobe ratio characteristics. We can perform two FFT transformations with two-dimensional FFT transformations at the same time. In order to reduce the amount of calculation, we can combine windowing and MTD window into one window, and only perform windowing once. Doppler phase compensation is processed in the one-dimensional pulse number. MTI is also carried out by the Doppler dimension. The MTI filter and the phase compensation window function can be synthesized together, and the MTI filtering and phase compensation can be performed at the same time. The improved time division MIMO radar signal processing flowchart is shown in Figure 4.

In the time division MIMO system working mode, the basic flow of echo signal processing is as follows:

1. The received echo signal is subjected to dechirp, moving target indication (MTI), and moving target detection (MTD) processing, mainly to eliminate clutter and stationary targets.
2. Simultaneous digital multibeam forming (receiving beamforming) is performed on the *M* echo signals of the receiving channel to obtain high-gain receiving beams pointing to *k* directions. We can flexibly control the direction of the receiving beam by setting the weighting factor of the receiving steering vector reasonably, so that the receiving beam can point to the space detection area we are interested in.

### 3.2. MIMO Algorithm GPU Parallel Processing Flow

The CUDA programming model is based on a heterogeneous system composed of CPU and GPU, so we not only optimize the execution efficiency of the device terminal code, but also take into account the efficiency of the collaborative work between the host and the device. The running process of CUDA program is as follows:

1. Allocate memory on the host and device and prepare data.
2. Copy data from the host to the device.
3. Start the kernel function for calculation.
4. Transmit the calculation result from the device to the host.

**Figure 5:**
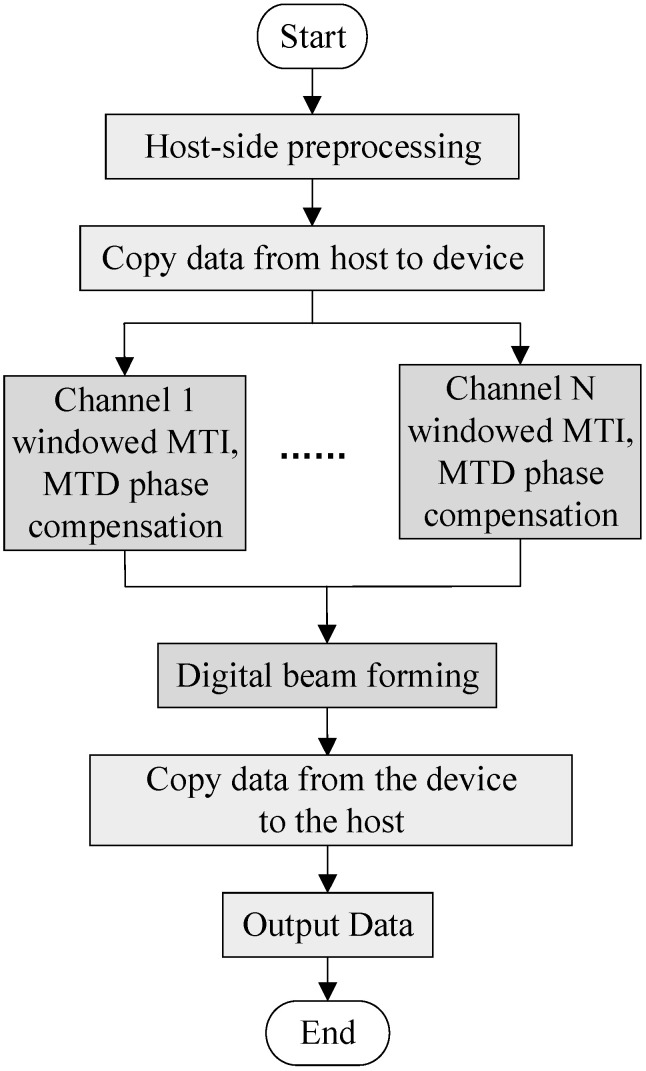
Improved time division MIMO radar GPU processing flowchart.

Time division MIMO radar signal processing simulation is a serial time process, in which different algorithms are processed sequentially according to the signal processing flow. However, the algorithm is still executed serially. Therefore, we can use GPU to perform parallel calculations. The fine-grained parallel strategy of time division MIMO radar signal processing simulation treats each algorithm as a computing node, and the entire algorithm is executed serially. Parallel execution improves the processing speed of the signal processing algorithm. The data in each channel of the time division MIMO radar are divided into threads and the data are processed at the same time. Figure 5 shows the CUDA implementation framework for accelerating the improved time division MIMO radar signal processing flow via GPU.

The fine-grained parallel simulation of time division MIMO radar signal processing based on CUDA not only conforms to the physical process of time division MIMO radar signal processing, but also makes full use of the hardware resources and computing power of GPU. This method is suitable for the time division MIMO radar signal processing flow with large data volume.

Using a MIMO radar with 4 transmitting elements and 50 receiving elements, the echo signals received after the four transmitting array elements transmitted are *T1*, *T2*, *T3*, and *T4*. The traditional time division MIMO radar processing process first performs windowing, MTI, MTD algorithms, Doppler phase compensation, and then beamforms the data of *T1*, *T2*, *T3*, and *T4*. The signal processing is greatly complicated, and it is difficult to meet the real-time requirements for processing with GPU, so the algorithm needs to be improved. In this paper’s process, windowing, MTI, MTD algorithm, and Doppler phase compensation are combined into one module for processing, which meets real-time requirements.

Firstly, preprocessing is performed on the CPU; then, the number of transmitting array elements, the number of receiving array elements, the number of distance units, the number of pulse points and other data are set, memory on the host is allocated, and the *T1*, *T2*, *T3*, *T4* receiving data and various window function data are imported into the memory for initialization. Memory is allocated on the device to copy the data on the host to the device. The downsampled data from the CPU side is copied from the CPU side to the GPU side. The echo signals *T1*, *T2*, *T3*, and *T4* copied to the GPU are copied into four copies, namely 1–16 copies of data. The main reason is that the speed measurement range of the original four data is too small. After copying 16 copies, the speed range can be expanded four times after compensation.

Then, the main algorithms are processed in parallel. The parallel processing of MIMO radar under the CPU/GPU architecture is mainly fine-grained parallel processing on the GPU. The specific processing of the *T1*’s data needs to be carried out on the GPU, and the *T1* part of the data must first be processed by the windowed MTI/MTD phase compensation module. *T2*, *T3*, and *T4* also perform the same operation.

Next is the beamforming module, which is processed on the GPU.

Finally, the data output operation is performed, the processed data is copied from the device to the host, the data is output finally, and the data processed by the entire time division MIMO radar signal is obtained for drawing a comparison.

#### 3.2.1. Preprocessing on the CPU

**Figure 6:**
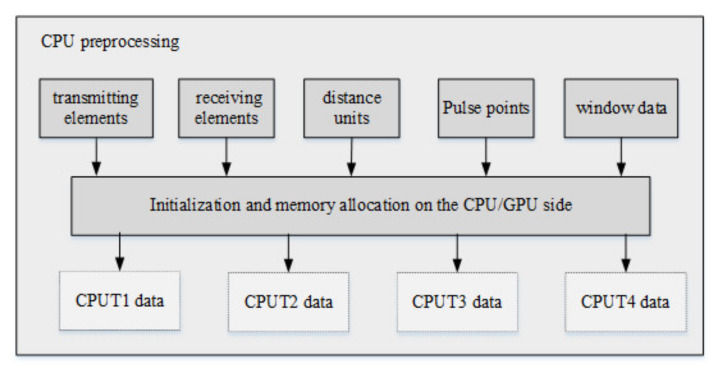
Flow chart of preprocessing on CPU.

The time division MIMO radar signal processing algorithm is preprocessed on the CPU firstly, including setting the number of transmitting array elements, the number of receiving array elements, the number of distance units, the number of pulse points, the received data and various window data. Then, it needs to allocate memory on the CPU and GPU, store the data on the CPU, and complete the preprocessing on the GPU. The flow of preprocessing on the CPU is shown in Figure 6.

#### 3.2.2. Windowed MTI/MTD Phase Compensation Module

**Figure 7:**
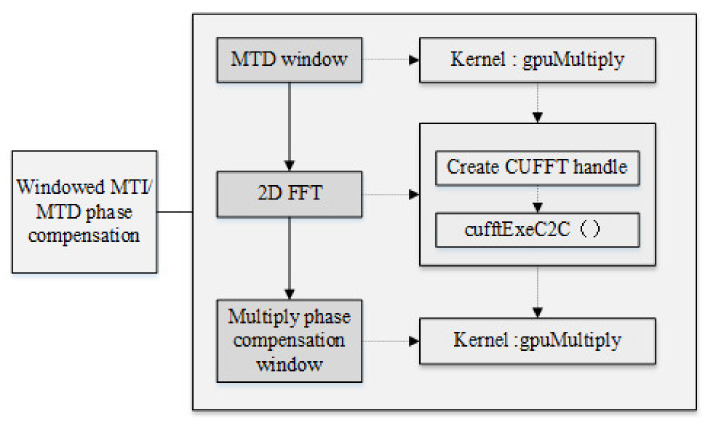
Windowed MTI/MTD phase compensation module diagram.

For a MIMO radar with 4 transmitting antenna array elements and 50 receiving array elements, 200 (4 × 50) virtual antenna array elements are generated, and all these signals need to be windowed. The specific implementation of the windowing operation can be converted to the frequency domain multiplication operation, so the CUFFT library provided by CUDA C is used to calculate the Fourier transform. The sampling data of the same distance unit of several adjacent pulse repetition periods in each frame are cancelled out by MTI in turn. MTD is used to operate FFT on all the sampling data of the same distance unit in each frame and carry out the same cancellation and FFT processing for all distance unit sampling points, so the MTI and MTD processing have correlation between periodic data. However, the sampling data of different distance units are not related. In this way, the MN two-dimensional matrixes obtained after pulse compression can be divided by the number of sampling points into N data blocks to achieve data-level parallelism between the echo data of different distance units, and each data block contains M pulses of the same distance unit echo data. In order to make the MTI cancellation effect better, a filter is used to achieve MTI. Since the target is a moving target with certain speed, Doppler phase compensation must be performed before beamforming. Figure 7 shows the block diagram of windowed MTI/MTD phase compensation module.

The specific operation can be divided into the following steps:

1. Use CUDA C to read MIMO radar echo data, extract all echo snapshot data in a certain CPI, and adjust the data format in conjunction with the usage specifications of the CUFFT library. It should be noted that the radar transmission signal data is stored in an array form to complete the data preparation; the data preparation stage is mainly data replication, which can be realized by using kernel functions.
2. Add filter window and MTD window to the echo data, mainly to perform dot multiplication on the data of *T1*, *T2*, *T3*, and *T4* with the window function that has been passed to the GPU.
3. Perform two-dimensional FFT transformation on the data after adding the MTD window function in the azimuth dimension and the number of sampling points, complete the MTI and MTD, and use the CUFFT library function to perform two-dimensional fast Fourier transform (FFT) on the data respectively.
4. Perform the phase compensation operation on the data after the two-dimensional FFT transformation, that is, perform the dot multiplication on the data and the preparation phase compensation window function, respectively. It should be noted that the added window function is different due to time delay of *T1*, *T2*, *T3*, and *T4*.

#### 3.2.3. Beamforming Module

**Figure 8:**
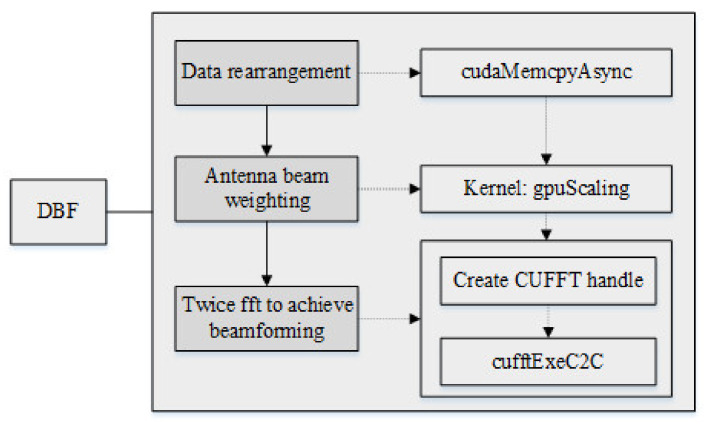
Block diagram of the beamforming module.

The DBF module needs to perform spatial filtering on the echo signal incident in a certain direction to obtain the echo data of the corresponding channel, such as the sum channel, the difference channel, and the auxiliary channel. For the antenna beam pointing at a certain moment, the weight vector of the spatial filter is multiplied by the incident echo signal to complete the DBF processing. When realizing full-wave position scanning, the calculation is performed in a circular manner. Each wave position is calculated once, and then the wave positions are changed in turn to ensure all wave positions are calculated cyclically. After completing the MTD of all virtual array elements, there are 200 virtual array elements. Each of them corresponds to 500 signals. At the same time, the digital beamforming operation can be regarded as the weighted summation of signals by 200 virtual array elements. Therefore, the realization of wave position digital beamforming can be regarded as the multiplication operation of the signal matrix and the array element weight matrix. The block diagram of the beamforming module is shown in Figure 8.

The specific operation can be divided into the following processes:

1. First, the data of *T1*, *T2*, *T3*, and *T4* are respectively compensated for the azimuth dimension Doppler phase, and the Doppler phase is canceled out.
2. Then, the data of the four channels *T1*, *T2*, *T3*, and *T4* are rearranged, and the data is transposed and rearranged.
3. Secondly, the data is multiplied by a two-dimensional DBF window function.
4. Finally, the data is subjected to a two-dimensional FFT to achieve a weighting effect, thus completing the entire beam-forming process.

### 3.3. Stream Acceleration Based on OpenMP

Due to the difference in computing power between CPU and GPU, CPU is used as the serial part, and GPU is used for the parallel part of real signal processing in traditional CPU/GPU computing. In this sense, this calculation is actually a GPU-based method. The main calculation task is executed by a large number of GPU threads, while the CPU threads are in a waiting state. The CPU can also participate in the parallel algorithm, the multicore parallel based on OpenMP and the GPU stream processing mechanism can be combined, and the CPU and GPU can be processed in parallel at the same time to improve the processing speed and meet the real-time requirements.

**Figure 9:**
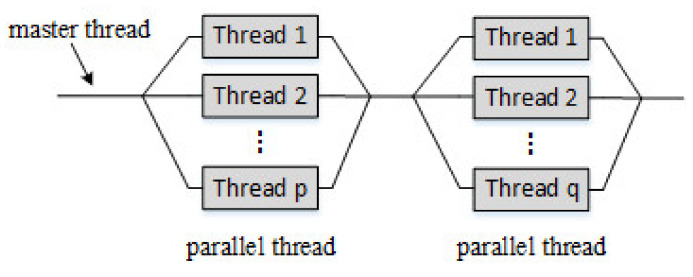
OpenMP’s fork-merge model.

OpenMP bifurcation-merge model: (1) OpenMP uses a fork-join model to achieve parallelization. (2) All OpenMP programs start from the main thread. The main thread executes serially until it encounters the first parallel region. (3) Bifurcation: After that, the main thread will create a group of parallel threads. (4) The code in the parallel region is surrounded by curly braces and then executed in parallel on multiple parallel threads. (5) Merging: After the parallel threads execute the code in the parallel region, they synchronize and end automatically, leaving only the main thread. (6) The number of parallel regions and the number of parallel threads can be arbitrary. The fork-merge model of OpenMP is shown in Figure 9.

The CUDA stream represents a GPU operation queue, and the operations in the queue are executed in the specified order. It can add some operations to the stream, such as kernel function startup, memory copy, etc. The order in which these operations are added to the flow is the order in which they are executed. Each stream can be viewed as a task on the GPU, and these tasks can be executed in parallel. When using CUDA stream, a device is selected that supports the device overlap function first. The GPU that supports the device overlap function can execute a CUDA core function while also performing data copy operations between the host and the device.

**Figure 10:**
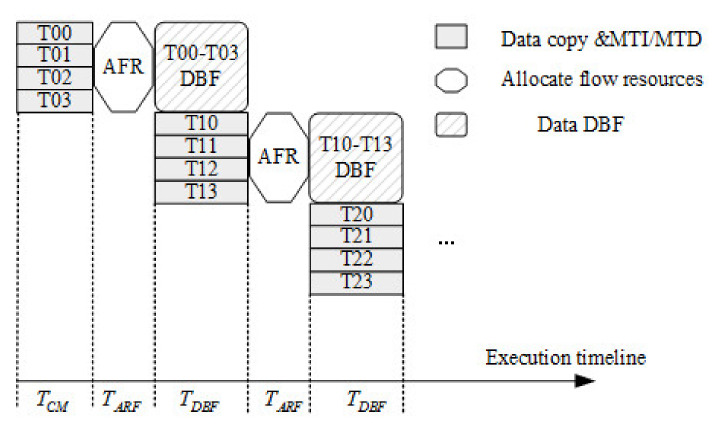
Stream parallel processing flow based on OpenMP.

In general, CPU memory is much larger than GPU memory. For large amounts of data, it is impossible to transfer the data in the CPU buffer to the GPU at one time. Therefore, it needs to be transferred in blocks. If one wants to perform kernel function operations on the GPU while transmitting in blocks, such asynchronous operations need to use the device overlap function to improve computing performance. However, there is no concept of flow in hardware. Instead, it contains one or more engines to perform memory copy operations and one engine to perform core functions. The stream acceleration parallel framework based on OpenMP is shown in Figure 10.

Running operations into the queue of the stream should be breadth-first rather than depth-first. In other words, instead of adding all operations of the first stream, and then adding all four operations of the second stream, the two streams are added alternately. Assuming that the copy operation takes time *a*, and the execution of the kernel function takes time *b*, then:

- When *a* ≈ *b*, the length of the timeline is about *4a*.
- When *a* > *b*, the length of the timeline is *4a*.
- When *a* < *b*, the length of the timeline is *3a* + *b*.

Stream parallelism can execute different kernel functions or pass different parameters to the same kernel function to achieve task-level parallelism. CudaMemcpy and CPU operations are synchronized. In order to achieve device overlap, CUDA provides cudaMemcpyAsync for data copy operations. It is asynchronous and executes the next step of the program without waiting for the copy to complete.

Coarse-grained parallel processing of time division MIMO radar signal processing flow on multicore CPUs uses OpenMP to copy data *T1*, *T2*, *T3*, and *T4*, windowing MTI/MTD modules, and DBF, totaling five parts to process in parallel. There are mainly three processing steps in the CPU/GPU cosimulation framework:

1. First, copy data *T00*, *T01*, *T02*, and *T03* from the CPU to the GPU, and perform MTI/MTD processing.
2. Open up five parallel threads on the CPU via OpenMP: the first parallel thread is responsible for processing the DBF algorithm of the previous data, and the second to the fifth threads are responsible for copying the data *T10*, *T11*, *T12*, and *T13* that will enter the GPU next time from the CPU to the GPU and undergo processing by MTI/MTD.
3. After the processing of step 2 is completed, the first parallel thread is responsible for the DBF algorithm of data *T10*, *T11*, *T12*, and *T13*. The second to fifth threads are responsible for copying the data *T20*, *T21*, *T22*, and *T23* that will enter the GPU next time from the CPU to the GPU and undergo processing by MTI/MTD. The processing is performed circularly as above.

When the first step data is being processed, the second step starts to load data, using ping-pong processing to reduce the time impact of copying data from the CPU to the GPU, so that the final signal processing meets the real-time requirements.

## 4. Experimental Results

In this paper, the data simulation of time division MIMO radar signal processing algorithm based on CPU/GPU parallel computing includes three improvements: the parallel of GPU-based radar processing algorithm, the improvement of time division MIMO radar signal processing algorithm, and the stream acceleration processing based on OpenMP. CPU, GPU acceleration and algorithm optimization improve the overall efficiency of the system. Four types of time division MIMO radar signal processing algorithm simulation experiments are designed: the time analysis of data copy, the parallelization of GPU-based radar processing algorithms, the improvement of time division MIMO radar signal processing algorithms, the impact of OpenMP-based stream acceleration processing on the simulation and the accuracy and error of the three methods are discussed, respectively.

In order to evaluate the experimental results, this article considers five groups of experiments with different data volumes, such as 960 × 500 × 200, 480 × 500 × 200, 240 × 500 × 200, 120 × 500 × 200, and 60 × 500 × 200. These experimental data are obtained via the actual measurement of the data acquisition card (DAQ). One Intel Xeon GOLD 5122 CPU (including 56 threads) and one NVIDIA Tesla T4 GPU (including 2560 cores) are used in the experiment. The simulation parameters and hardware specifications are shown in Table 1 and Table 2. In addition, the software environment consists of four parts. Specifically, the operating system is Windows Server 2018, the Intel C++ writer is Visual Studio 2013, and CUDA 10.1 is selected to drive GPU parallel computing. In addition, OpenMP is used for thread parallel processing, and its number can be set according to the number of CPU cores in a specific device. There are five CPU task-level threads for parallel processing, and each time division MIMO radar signal processing algorithm opens up GPU threads for parallel computing. In the collaborative computing mode, the running time of the Matlab code only considers the time of the core time division MIMO radar algorithm. The running result of the GPU code takes into account the input and output time, including GPU memory allocation and data transmission between CPU and GPU, and core GPU time division MIMO radar algorithm time [25].

### 4.1. Data Copy Time Analysis

From the execution of the CUDA kernel function, it can be seen that the execution of the kernel function first needs to store data from the memory to the video memory, which takes data transmission time; then reads the data from the video memory and uses multiple threads to process the data, which takes calculation time. Therefore, the time consumed to execute the algorithm is the sum of the data transmission time and the calculation time. Before analyzing the signal processing algorithm, it is necessary to analyze the data transmission time.

**Figure 11:**
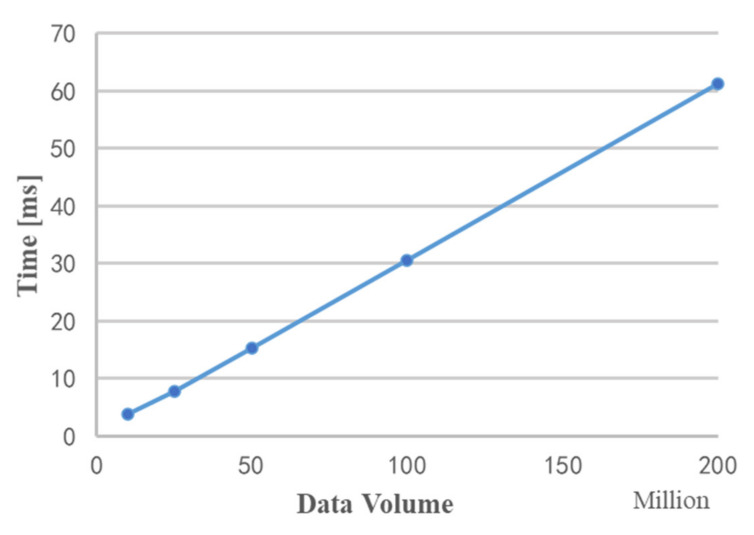
Time chart for data copy.

Figure 11 shows the time required for data copy under different data volumes. It can be seen from the figure that as the data volume increases, the copy time and data volume show a linear growth trend.

It is worth noting that although this article is divided into modules to study the implementation of parallel acceleration of MIMO radar echo signal processing, it is not necessary for each module to copy data from the video memory to the memory after execution. When the video memory is enough to store the input data of all the operations, there is no need to take the data out of the video memory after each module is executed; it is only needed to release the unnecessary video memory variables in time.

### 4.2. GPU-Based Time Division MIMO Radar Signal Processing Algorithm Analysis 

The accuracy of simulation analysis and runtime are two important factors for GPU acceleration. Therefore, we mainly analyze the MIMO radar signal processing flow in two aspects. The first is the analysis of the accuracy of GPU accelerated simulation. Consider the data results of each algorithm node, compare the GPU calculation results with the original Matlab simulation results, and verify the correctness of the simulation via error analysis. The second is the analysis of performance improvement of GPU acceleration, mainly by selecting data of different data volumes for processing and analyzing the acceleration ratio of GPU via its runtime results.

#### 4.2.1. Analysis of the Accuracy of GPU Accelerated Simulation

**Figure 12:**
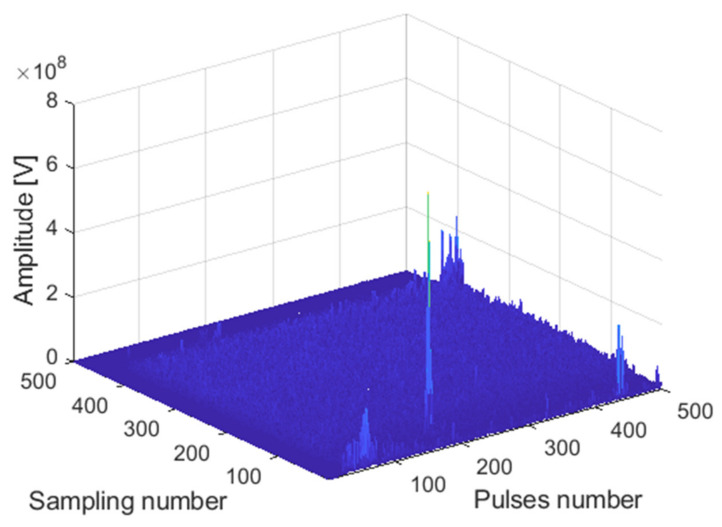
Matlab DBF result graph.

**Figure 13:**
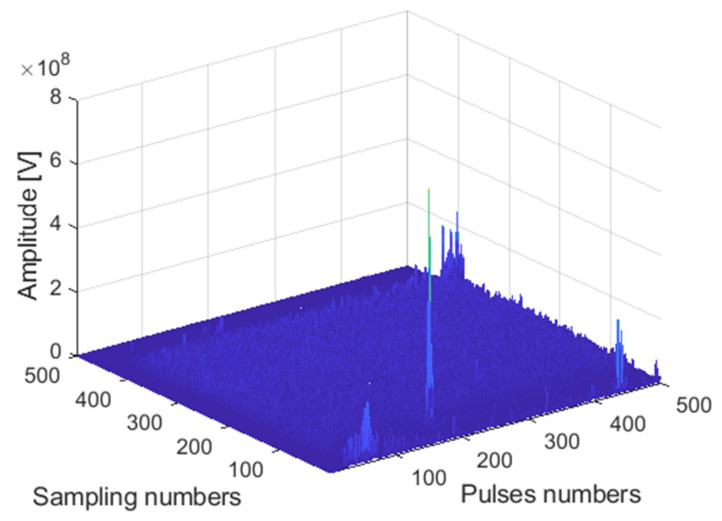
GPU DBF result graph.

**Figure 14:**
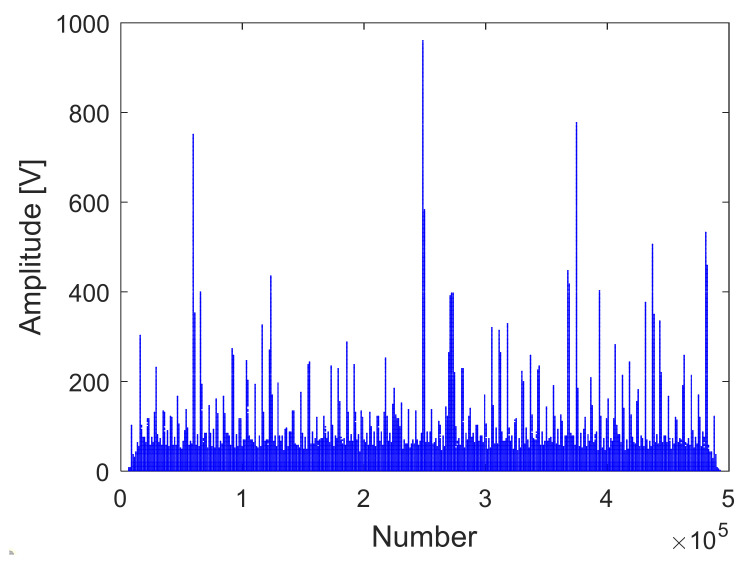
GPU and Matlab data error statistics.

The receiving channel is randomly selected, taking the *45th* receiving channel as an example. As shown in Figure 12, Figure 13 and Figure 14, there are the final output results of the GPU-based time division MIMO radar signal processing algorithm, the output results of Matlab, and their error statistics, respectively.

As can be seen from the above figures, the Matlab results are consistent with the GPU results. The expected value of multiple data error statistics in Figure 14 is 106.56, and the mean square error (MSE) is 128.69, namely, error magnitude is about 10^2^. The specific cause of the error is because the use of float to store data will cause an error between the true value and stored data. The binary storage digits after the decimal point of the float type are 21 digits, which is 10^6^, and the storage accuracy is 6 digits after the decimal point. However, the magnitude of the data is about 10^8^, and the magnitude of the error is about 10^2^. The result of dividing the magnitude of the error by the magnitude of the data is roughly about 10^−6^. The error statistics results basically meet the float error range, which verifies the correctness of the GPU acceleration results.

#### 4.2.2. Analysis of GPU Accelerated Performance Improvement

The MIMO radar signal processing algorithm mainly includes three parts: windowing processing, MTI/MTD processing, and beam-forming processing. We mainly choose different data volumes for these three algorithms and analyze the impact of different data volumes on the three algorithms. The acceleration ratio of GPU processing is analyzed by Matlab runtime and GPU runtime data volume.

Table 3 shows the influence of different data volumes on the three algorithms. It can be seen that the simulation time is increasing as the data volume increases. The data copying time is fixed, and it is difficult to accelerate the processing. The windowing and MTI/MTD algorithm account for about half of the total time, so optimization can be considered. Due to the limitation of the number of Tesla *T4* cores, when the amount of data reaches a certain level, the acceleration is limited. As the data needs to be rearranged in the DBF algorithm, which includes the time of data movement, it takes a long time.

**Figure 15:**
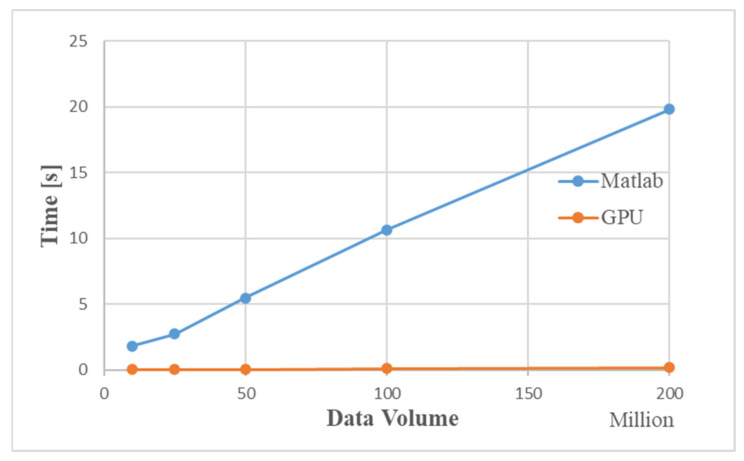
Comparison of time used by GPU and Matlab.

Table 4 shows the GPU processing flow and the schedule used by Matlab under different data volumes. Figure 15 is a time comparison chart. The simulation results show that as the amount of data increases, the simulation time for Matlab to process data increases significantly. When the data volume is about 200 million, the GPU-based MIMO radar signal processing algorithm is 100 times faster than the traditional CPU processing method.

### 4.3. Improved Time Division MIMO Radar Signal Processing Algorithm Analysis Based on GPU

The GPU-based improved time division MIMO radar signal processing simulation analysis is the same as the ordinary GPU-based time division MIMO radar simulation analysis. It is divided into the analysis of the accuracy of GPU accelerated simulation and the analysis of performance improvement of GPU acceleration.

#### 4.3.1. Analysis of the Accuracy of GPU Accelerated Simulation

**Figure 16:**
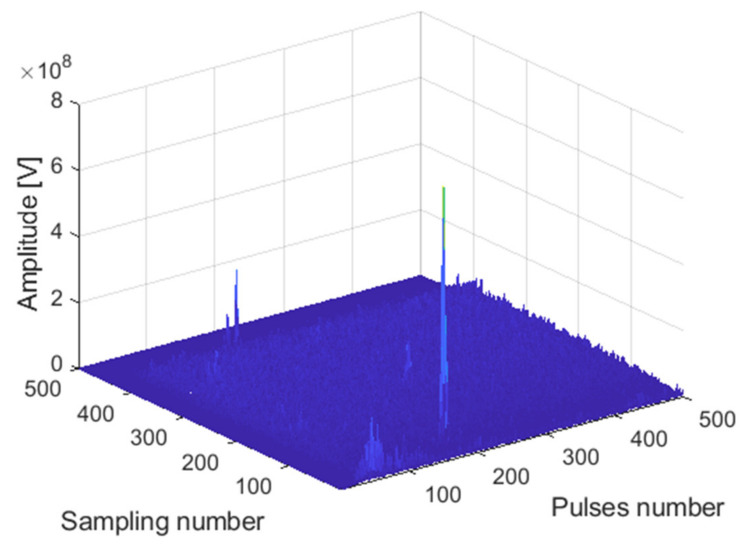
Matlab DBF result graph.

**Figure 17:**
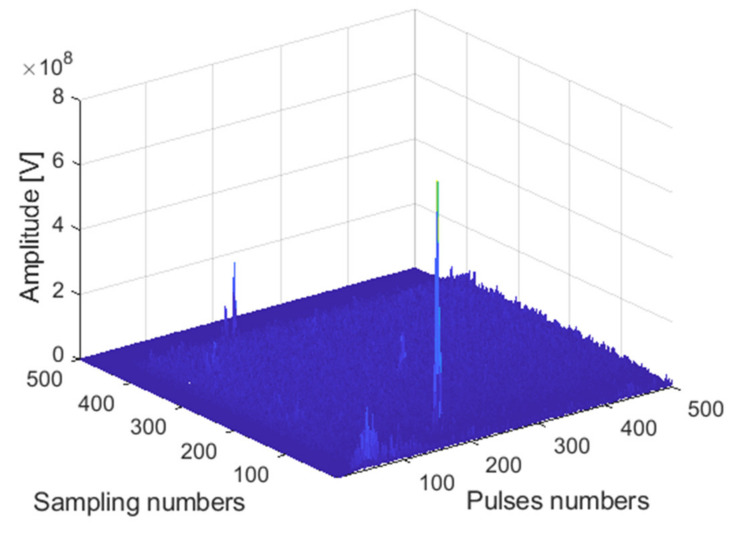
GPU DBF result graph.

**Figure 18:**
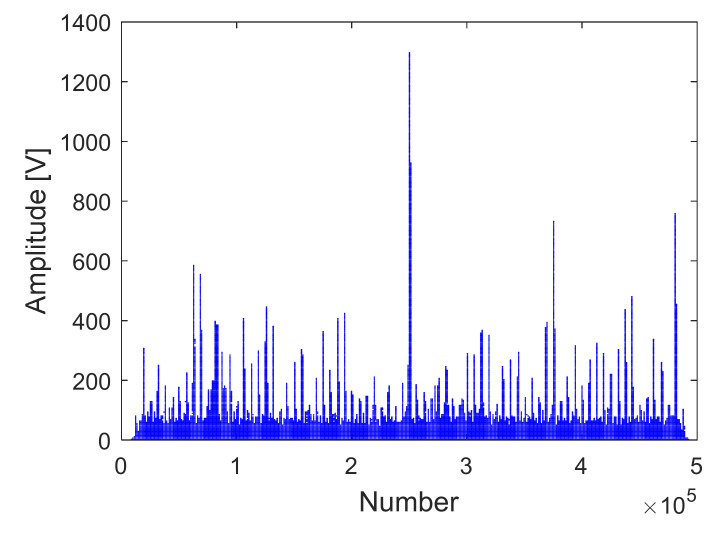
Data error statistics of GPU and Matlab.

The receiving channel is randomly selected, taking the *75th* receiving channel as an example. As shown in Figure 16, Figure 17 and Figure 18, there are the final output results of the GPU-based time division MIMO radar signal processing algorithm and the output results of Matlab and their error statistics, respectively.

The expected value of multiple data error statistics in Figure 18 is 120.87, and the mean square error (MSE) is 158.12, namely, error magnitude is about 10^2^. While the magnitude of the data is about 10^8^, the result of dividing the magnitude of the error by the magnitude of the data is roughly about 10^−6^. The error statistics results meet the float error range. As can be seen from the above figures, the Matlab results and the GPU results are almost identical, which verifies the correctness of the GPU acceleration results.

#### 4.3.2. Analysis of GPU Accelerated Performance Improvement

The improved MIMO radar signal processing algorithm mainly includes two parts: windowing MTI/MTD processing and beamforming processing.

Table 5 shows the impact of different data volumes on the two algorithms. It can be seen that the time of DBF is similar to the time used in Table 4, and the time of windowing MTI/MTD is reduced compared with the time of the first two algorithms in Table 4.

**Figure 19:**
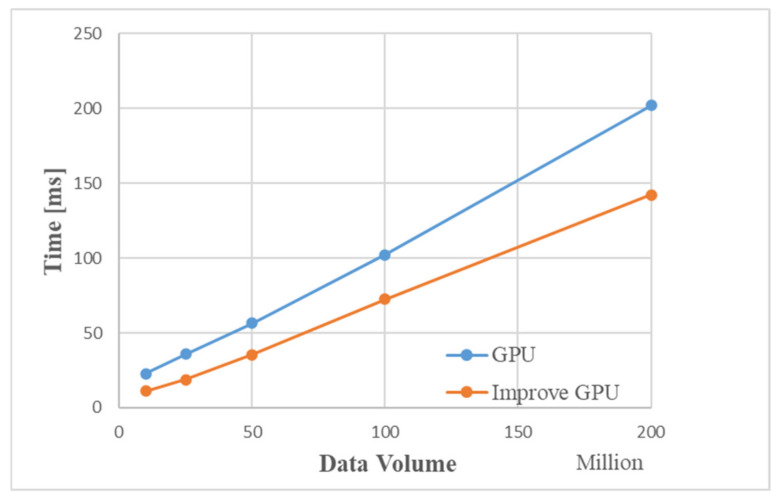
Comparison of the time taken by the GPU with the improved algorithm and the traditional GPU.

Figure 19 is a comparison graph of the time by the improved GPU processing flow and the traditional GPU. The simulation results show that the improved MIMO radar signal processing algorithm based on GPU is about 50 ms faster than the traditional GPU processing method. When the data volume is about 200 million, the processing time of the improved GPU processing flow is about 150 ms, and the entire processing flow of Matlab takes 19.807 s. Compared with Matlab processing, the improved GPU algorithm is 130 times faster.

### 4.4. Stream Acceleration Based on OpenMP

The OpenMP-based stream acceleration processing is based on the original improved algorithm, and the data copy time and data calculation time are processed in parallel. The results of the operation shown in Figure 16, Figure 17 and Figure 18 have the same results, verifying the correctness of the simulation results. We mainly analyze the improvement in processing speed.

**Figure 20:**
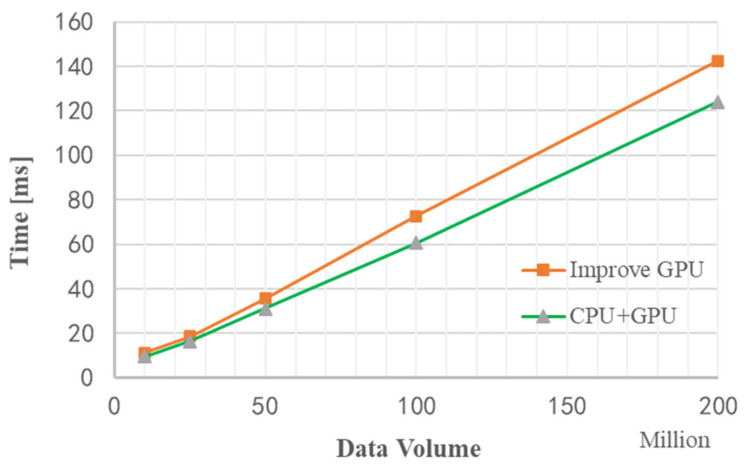
Comparison of GPU time spent on stream acceleration processing and improved algorithm.

As shown in Figure 20, it is the total time (including data copy time and data calculation time) used for OpenMP-based stream acceleration processing under different data volumes. When the data volume is about 200 million, the total time is saved by 20 ms, which verifies the performance improvement of stream acceleration processing.

**Figure 21:**
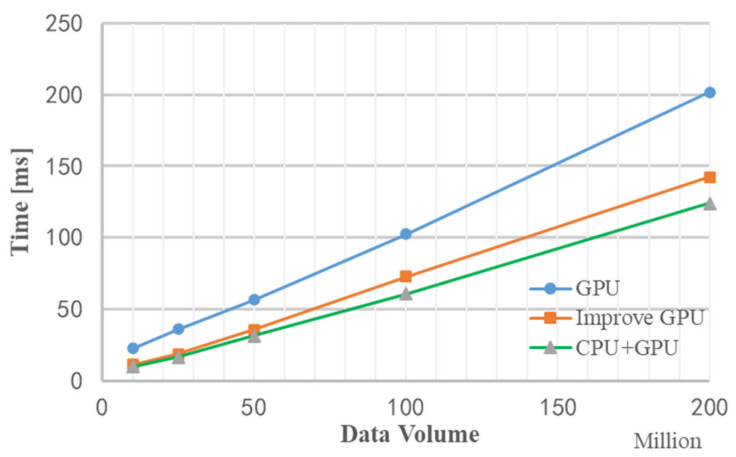
Comparison of the time used by the three acceleration methods.

Figure 21 is a graph of the runtime of three methods without improved GPU processing, improved GPU processing, and stream acceleration processing under different data volumes. It can be seen that when the amount of data is relatively small, the performance of stream acceleration is not well reflected, but as data volume increases, the advantages of stream acceleration processing appear. When the data volume is about 200 million, the stream acceleration processing method using CPU+GPU is about 20 ms faster than the improved GPU processing method. The entire processing flow of Matlab takes 19.807 s, which is about 150 times faster than Matlab processing using the stream acceleration processing method based on CPU+GPU.

## 5. Conclusions

This paper uses CPU/GPU computing technology to solve the calculation bottleneck problem of the traditional time division MIMO radar signal processing algorithm. A parallel simulation method for time division MIMO radar signal processing based on CPU/GPU parallel is proposed. Specifically, this article introduces three improvements: First, the GPU-based time division MIMO radar signal processing method, which greatly improves the computing power of time division MIMO radar signal processing and promotes the possibility of real-time processing of time division MIMO radar signals. The second is to propose an improved time division MIMO radar signal processing algorithm, which combines part of the algorithm content, which speeds up the processing speed from the original algorithm; the third is to join the OpenMP-based stream parallel processing method, parallel calculation by distributing data copy and data calculation into different streams, which further improves the simulation efficiency, and on the basis of the original GPU method, a desirable acceleration effect has been achieved, which basically meets the requirements of real-time processing.

The experimental results show that the GPU-based time division MIMO radar signal processing method has increased the processing efficiency of single-core CPU by more than 50 times compared with the classic Matlab CPU method, and the GPU calculation of the improved algorithm has reached a speedup of 130 times. In addition, compared with classic GPU processing, the performance of OpenMP-based stream acceleration processing has increased by 20%. This method improves the simulation efficiency and has the advantages of energy saving and low hardware cost. This method is suitable for low-altitude time division MIMO radar signal processing simulation. Because MIMO radar signal processing has the characteristics of large data volume, it is expected to be better applied in multiantenna target detection. The future work of this research will form a complete set of methods based on time division MIMO radar signal processing and target detection, and target information extraction; using multi-GPU and CPU/GPU collaborative computing methods to apply to MIMO radar signal processing and target recognition, which will be a preliminary attempt made for the real-time processing and widespread application of MIMO radar products.

## Figures and Tables

**Table 1:** Simulation parameters.

Parameters	Value
Wave length	0.285 m
PRF	1000 Hz
Pulse width	240 μs
Band width	15 MHz
Sampling rate	4 MHz
Emitting array element number	4
Receive array elements	64

**Table 2:** Hardware specifications.

Parameters	Value
Number of CUDA cores	2560
GPU float performance	8.1 TFlops
Dedicated GPU memory	16 GB
GPU memory bandwidth	320 GB/s
GPU frequency	1.59 GHz
Number of CPU cores	28 × 2
Receive array elements	64

**Table 3:** Simulation time (ms).

Data Volume	Copy	Window	MTI/MTD	DBF
60 × 500 × 50	3.85219	6.84518	7.09174	4.83832
120 × 500 × 50	7.72541	8.5632	12.50515	7.1352
240 × 500 × 50	15.3108	11.3492	17.77053	11.9628
480 × 500 × 50	30.5783	16.9038	32.03888	22.8164
960 × 500 × 50	61.18	32.4944	63.9504	44.254

**Table 4:** Simulation time comparison.

Data Volume	Matlab [s]	GPU [ms]
60 × 500 × 50	1.840	22.62743
120 × 500 × 50	2.736	35.92896
240 × 500 × 50	5.462	56.39333
480 × 500 × 50	10.638	102.33738
960 × 500 × 50	19.807	201.8788

**Table 5:** Simulation time (ms).

Data Volume	Window	MTI/MTD	DBF
60 × 500 × 50	3.85219	2.48733	4.93517
120 × 500 × 50	7.72541	3.95418	7.0279
240 × 500 × 50	15.3108	8.4071	11.874
480 × 500 × 50	30.5783	19.2561	22.7552
960 × 500 × 50	61.18	36.9909	44.2316

## Data Availability

Not applicable.
